# A cultural side effect: learning to read interferes with identity processing of familiar objects

**DOI:** 10.3389/fpsyg.2014.01224

**Published:** 2014-10-31

**Authors:** Régine Kolinsky, Tânia Fernandes

**Affiliations:** ^1^Fonds de la Recherche Scientifique-FNRSBrussels, Belgium; ^2^Unité de Recherche en Neurosciences Cognitives, Center for Research in Cognition and Neurosciences, Université Libre de BruxellesBrussels, Belgium; ^3^Faculty of Psychology, Center for Research in Psychology, Universidade de LisboaLisboa, Portugal

**Keywords:** visual object recognition, mirror images, enantiomorphy, literacy

## Abstract

Based on the *neuronal recycling hypothesis* (Dehaene and Cohen, 2007), we examined whether reading acquisition has a cost for the recognition of non-linguistic visual materials. More specifically, we checked whether the ability to discriminate between mirror images, which develops through literacy acquisition, interferes with object identity judgments, and whether interference strength varies as a function of the nature of the non-linguistic material. To these aims we presented illiterate, late literate (who learned to read at adult age), and early literate adults with an orientation-independent, identity-based same-different comparison task in which they had to respond “same” to both physically identical and mirrored or plane-rotated images of pictures of familiar objects (Experiment 1) or of geometric shapes (Experiment 2). Interference from irrelevant orientation variations was stronger with plane rotations than with mirror images, and stronger with geometric shapes than with objects. Illiterates were the only participants almost immune to mirror variations, but only for familiar objects. Thus, the process of unlearning mirror-image generalization, necessary to acquire literacy in the Latin alphabet, has a cost for a basic function of the visual ventral object recognition stream, i.e., identification of familiar objects. This demonstrates that neural recycling is not just an adaptation to multi-use but a process of at least partial exaptation.

## Introduction

According to several theories concerning the functional organization of the brain, it is quite common for neural circuits established for one purpose to be *exapted* (Gould and Vrba, 1982) or *tinkered* (Jacob, 1977) during evolution (e.g., the *massive redeployment* hypothesis, Anderson, 2007a,b) or normal development (the *neuronal recycling* hypothesis, Dehaene and Cohen, 2007; Dehaene, 2009), so that they may come to serve a different purpose (see Anderson, 2010, for a review). The neuronal recycling hypothesis is specifically interested in the acquisition of cultural inventions such as reading or mathematics that have emerged too recently in mankind, precluding evolution to have engendered cortical circuits dedicated to these purposes. Consequently, these cognitive abilities have to be learned and must find their *neuronal niche*, namely pre-existing neural systems “that are sufficiently close to the required function and sufficiently plastic as to reorient a significant fraction of their neural resources to this novel use” (Dehaene and Cohen, 2007, p. 384).

Under this hypothesis, cultural learning is generally facilitated by pre-existing cortical properties. In the case of reading acquisition, several characteristics of the ventral visual pathway, including the general properties for invariant object recognition (e.g., Serre et al., 2007; Ullman, 2007), may explain why a subpart of the left ventral visual system, termed the visual word form area (*VWFA*, e.g., Cohen et al., 2000), has been partially co-opted or *recycled* for recognizing the visual shapes of written symbols.

However, it is quite unlikely that all pre-existing cortical properties suit the new, target function. In some cases the acquisition of cultural inventions may require the overcoming of properties that were useful for the original function, but are deleterious for the new one. An example of such an undesirable property for reading acquisition is *mirror-image generalization*, also called *mirror invariance*, namely the tendency to confuse lateral reflections.

Difficulties in differentiating and remembering lateral reflections or *enantiomorphs* have been reported in infants (e.g., Bornstein et al., 1978; Bornstein, 1982), children (e.g., Gibson et al., 1962; Rudel and Teuber, 1963; Cronin, 1967; Gibson, 1969; Casey, 1984; Shepp et al., 1987; de Kuijer et al., 2004), and even adults (e.g., Butler, 1964; Sekuler and Houlihan, 1968; Standing et al., 1970; Wolf, 1971; Farrell, 1979; Nickerson and Adams, 1979; Martin and Jones, 1997; de Kuijer et al., 2004; Rentschler and Jüttner, 2007), for whom long-term priming (with primes and probes separated by several minutes) is unaffected by left-right reflection (e.g., Biederman and Cooper, 1991; Stankiewicz et al., 1998; Fiser and Biederman, 2001). Mirror invariance seems to have been deeply rooted by evolution into the visual system: many animals (e.g., fishes, octopuses, rodents, and monkeys) are also confused by enantiomorphs (e.g., Sutherland, 1960; see a review in, e.g., Corballis and Beale, 1976), and neurons in the monkeys' inferotemporal cortex generalize over mirror reversal (Logothetis and Pauls, 1995; Logothetis et al., 1995; Rollenhagen and Olson, 2000; Baylis and Driver, 2001).

This characteristic of the visual system presumably arose in the course of evolution because most natural visual categories are invariant across enantiomorphic changes (Corballis and Beale, 1976; Gross and Bornstein, 1978), and hence, lateral reversals convey little information about the object viewed: “a tiger is equally threatening when seen in right or left profile” (Rollenhagen and Olson, 2000, p. 1506). However, whereas useful for the recognition of natural objects, mirror invariance is deleterious for reading in the Latin alphabet. As this script includes minimal mirror pairs such as *b* and *d*, mirror generalization would impede reading acquisition, leading to confusions between mirrored letters. Mirror invariance is an intrinsic property of a subpart of the visual cortex that has thus to be unlearned or at least suppressed so that one can become a fluent reader.

Consistently, in fluent adult readers the VWFA simultaneously shows a maximal effect of mirror priming for pictures of familiar objects, fruits, or animals and an absence of mirror priming for words (Dehaene et al., 2010a) and letters (Pegado et al., 2011). In an orientation-independent task in which participants had to judge either whether a target was larger or smaller in real-life than a standard computer screen (Dehaene et al., 2010a) or whether it stayed (or not) within a central frame (Pegado et al., 2011), each target being preceded by either the same or a different prime that appeared either in the same orientation or mirrored, repetition suppression (i.e., decreased fMRI activation due to processing subsequent stimuli with identical attributes) was observed in the VWFA only for mirrored pictures, not for mirrored words or letters. In addition, in Dehaene et al. (2010a), the size judgments were accelerated by mirrored primes much more for pictures than for words.

At the behavioral level, there is considerable evidence for a progressive unlearning of mirror invariance in children, and this process, crucial for linguistic materials, generalizes to non-linguistic stimuli (e.g., Gibson et al., 1962; Rudel and Teuber, 1963; Cronin, 1967; Gibson, 1969; Serpell, 1971; Casey, 1984). These developmental studies confounded age with literacy level, leading to the view that the ability to discriminate mirror images would mainly depend on neural maturation (e.g., Orton, 1937; Corballis and Beale, 1976; Casey, 1984). However, more recent work on adults disentangled the influence of literacy from that of neural maturation. In these studies, adults who remained illiterate for strictly socioeconomic reasons were far poorer at discriminating between non-linguistic enantiomorphs (of geometric or blob-like shapes, as well as of pictures of familiar objects like tools, furniture, and clothes) than both *early literates*, who learned to read at school in childhood, and *late literates*, who never attended school in childhood but learned to read in adulthood in special literacy classes (Kolinsky and Verhaeghe, 2011; Kolinsky et al., 2011; Fernandes and Kolinsky, 2013). Therefore, it is not neural maturation, but the need to take enantiomorphic contrasts into account when learning a script that includes mirrored symbols that pushes one to unlearn (Dehaene et al., 2010a) or at least partly inhibit (Duñabeitia et al., 2011; Perea et al., 2011) mirror-image generalization during explicit, conscious processing of both linguistic and non-linguistic materials.

In readers, this unlearning process may have adverse consequences for object recognition if objects vary by orientation in a way irrelevant to the task. Consistent with this idea are the priming effects observed by Dehaene et al. (2010a) in the size judgment task: for pictures of objects, behavioral priming effects were smaller for mirrored than for identical primes. Similarly, in a behavioral orientation-independent, identity-based same-different comparison task in which participants had to respond “same” to both physically identical and mirror images, Dehaene et al. reported that participants showed interference from irrelevant mirror variations (henceforth, *mirror interference*): they were faster to respond to identical than to mirrored images of non-linguistic objects. Using a similar identity-based task, Pegado et al. (2014) provided direct evidence supporting the idea that such mirror interference is a side effect of literacy acquisition: both early and late literate adults presented slowed responses and increased error rates when letters strings, false-fonts, and pictures of familiar objects were mirrored rather than strictly identical, whereas illiterate adults did not present any cost for mirrored pairs.

In the present study, we also compared illiterate, late literate and early literate adults, using an identity-based same-different comparison task similar to the one used by Dehaene et al. (2010a) and Pegado et al. (2014): in two experiments, on each trial participants were asked to decide whether the second stimulus (*S2*) was the same or not as the first one (*S1*), independently of its orientation. Our aim was two-fold.

First, we checked for the specificity of the literacy effect reported by Pegado et al. (2014) by comparing the mirror interference effect to the interference caused by another orientation contrast, i.e., rotations in the image plane or *plane rotations* (henceforth, *rotation interference*). As already noted by Gibson et al. (1962), both mirror images and plane rotations distinguish graphic forms in the Latin alphabet (e.g., d—b, and d—p, respectively). Literacy would thus impact on the ability to discriminate both types of orientation contrasts. Yet, according to the neuronal recycling hypothesis (Dehaene, 2009), the impact of reading acquisition should be stronger on enantiomorphy, as the ventral visual pathway is originally sensitive to plane rotations but not to mirror images (e.g., Logothetis and Pauls, 1995; Logothetis et al., 1995). Consistently, in orientation-dependent tasks, both illiterate and literate adults explicitly discriminate plane rotations far more easily than enantiomorphs (Kolinsky et al., 2011; Fernandes and Kolinsky, 2013). It is thus probable that in an identity-based task, (irrelevant) plane-rotation contrasts would be more automatically activated than (irrelevant) mirror-image contrasts. Although this difference might hold true for all participants, whatever their literacy level, it might be particularly strong for illiterates, as they display very poor enantiomorphic discrimination (Kolinsky and Verhaeghe, 2011; Kolinsky et al., 2011; Fernandes and Kolinsky, 2013). Here, we thus predicted that the interference effect would be stronger with plane rotations than with mirror images for all participants, and that rotation interference would be less modulated by literacy than mirror interference, which was expected to be far stronger in literate than illiterate participants, as was the case in Pegado et al. (2014).

Second, we checked whether the strength of the interference displayed by the participants would vary as a function of the nature of the non-linguistic material. Across the two experiments, we examined the impact of familiarity of the material. In Experiment 1, on familiar objects, we also examined the role of graspability, namely of the degree by which visuomotor information is critical to the representation of the object, by comparing identity-based judgments for *non-graspable* and *graspable* objects; for the latter (e.g., a hammer), there is a strong relationship between shape and manner of being grasped or manipulated.

The impact of familiarity of the material was examined by comparing pictures of familiar objects (Experiment 1) to geometric shapes (Experiment 2). We predicted that interference from irrelevant orientation variations would be stronger with geometric shapes than with familiar objects (at least with non-graspable ones), for both mirror images and plane rotations. This prediction is based on three non-mutually exclusive reasons. First, simple geometric shapes may be more similar to letters than familiar objects, and there seems to be an early bias in the VWFA for processing visual features of symbol-like shapes. In support of this idea, Szwed et al. (2011) found that configurations of line junctions, which seem universally used in writing systems worldwide (Changizi et al., 2006; but see discussions in Coltheart, 2014; Dehaene, 2014; Downey, 2014), specifically promote activation in the ventral fusiform part of the visual system. As mirrored letters or words are much more differentiated in the VWFA than mirrored pictures (Dehaene et al., 2010a; Pegado et al., 2011), if geometric shapes were treated as visual features of symbol-like shapes, then their mirror images would also be more differentiated than mirrored familiar objects, hence leading to stronger mirror interference for geometric shapes in an identity-based task. An early bias to the processing of this kind of material might also explain that even in for 4-year-old preliterates, letter-like shapes already activate the VWFA (Cantlon et al., 2011). In addition, even young preliterate children and illiterate adults may benefit from minimal exposure to letters and other symbols. Consistently, illiterate adults with some knowledge of letters already process letters differently than non-letter stimuli (Fernandes et al., 2014). Finally, according to some visual models, novel shapes are coded in a viewpoint-dependent, orientation-specific way, whereas familiar objects (especially non-graspable ones) benefit from viewpoint-independent, object-centered representations (e.g., Tarr and Bülthoff, 1993). The enantiomorphic performance of illiterate adults is consistent with all these views: in an orientation-dependent task requiring explicit discrimination of mirror images, their performance was facilitated for geometric shapes compared to (non-graspable) familiar objects (Fernandes and Kolinsky, 2013). Here, we thus expected all groups to present more mirror and rotation interference with geometric shapes than with familiar objects.

Our former work using an orientation-dependent task also showed that enantiomorphic performance was modulated by the graspability of familiar objects (Fernandes and Kolinsky, 2013). Action-related information seems to be automatically invoked by graspable objects like tools, even when there is no action on the object, as in passive viewing or perceptual tasks (e.g., Tucker and Ellis, 1998; Creem-Regehr and Lee, 2005). Fernandes and Kolinsky (2013) manipulated specifically whether the position of the object in the picture signaled the use of one particular hand if one would want to grasp it. Although no overt grasping response was required, enantiomorphic performance was facilitated for graspable compared to non-graspable objects, i.e., those for which the position of the object does not signal the use of one particular hand. This was the case in all groups (illiterate, late and early literate adults) and probably reflects that orientation signals the visuomotor properties of graspable objects, for which these properties are critical but not to non-graspable ones (Murata et al., 2000; Valyear et al., 2006; Rice et al., 2007). Therefore, in Experiment 1, we compared graspable to non-graspable familiar objects, predicting that mirror interference would be stronger with graspable than non-graspable objects.

Since the identity judgment used in the present study is an easy task, even for unschooled illiterates (cf. Pegado et al., 2014), instructions emphasized both accuracy and speed, with the latter being the principal measure of interest. For both accuracy and response times (*RTs*), we compared performance on physically identical trials, in which both object identity and orientation were the same, to performance on different-orientations trials, in which object identity was also the same but S2 was either a mirror image or a plane rotation of S1. Yet, since we know that illiterates have difficulties at speeded responses, to which they are not used to (e.g., Morais and Kolinsky, 2002; Ventura et al., 2007; Kolinsky et al., 2011), and since they often present quite variable performance (e.g., Kolinsky et al., 2011), we expected them to display slower and perhaps less accurate responses than literates. To control for this overall between-group difference, as in Pegado et al. (2014) we used a normalized *interference index* computed, separately for mirror and for plane-rotation variations, as the ratio between the RT (or accuracy) difference between different-orientation and identical trials, using as denominator the sum of RTs (or accuracy) on different-orientation and identical trials. We predicted that both late and early literates would present stronger interference from irrelevant orientation variations than illiterates, especially with enantiomorphs.

## Experiment 1: identity judgments on familiar objects

### Method

#### Participants

Forty-nine adults were paid for their participation to a larger battery of tests, including orientation-dependent tasks using the same materials (see below). According to their schooling and literacy levels (see below), they were assigned to three groups: unschooled illiterates, unschooled late literates, and schooled early literates. The ethical committee of the Psychological and Educational Sciences Faculty at Université Libre de Bruxelles approved the study protocol; all participants provided oral informed consent.

To check for task commitment, we first examined the *Signal Detection Theory* (SDT) *d′* statistic adapted for same-different comparison tasks (Macmillan and Creelman, 2005), considering as *hits* the correct “different” responses on trials in which both object identity and orientation were different, and as *false alarms* the incorrect “different” responses on identical trials, in which both object identity and orientation were the same (see mean correct scores in Table 1, separately for each group). Two illiterates were excluded from further analyses because they probably have not understood the task: both presented a *d*′ of zero, while all other participants were quite able to perform the task with mean *d*′ scores of 4.36 (*SD* = 1.56), 5.74 (*SD* = 1.11), and 6.01 (*SD* = 0.67) for illiterates, late literates and early literates, respectively.

**Table 1:** **Experiment 1: Mean performance in the identity-based same-different comparison task for familiar objects, presented by object type, trial type, and group of participants**.

	**Trial type**		**Graspable objects**			**Non-graspable objects**		
	**Expected response**	**Orientation**	**Illiterates**	**Late literates**	**Early literates**	**Illiterates**	**Late literates**	**Early literates**
Accuracy (%)	Different		84.57 [13.86]	94.49 [5.83]	96.09 [4.33]	86.67 [13.71]	95.42 [5.26]	97.02 [2.83]
	Same	Identical	87.18 [10.01]	95.93 [4.56]	96.67 [3.37]	86.06 [10.81]	94.27 [7.14]	97.13 [2.53]
	Same	Mirror	86.82 [9.01]	95.47 [4.00]	96.67 [2.74]	87.18 [8.82]	95.27 [4.08]	96.40 [3.11]
	Same	Rotation	89.00 [7.78]	94.53 [5.90]	97.00 [2.17]	87.24 [10.16]	94.47 [4.60]	94.47 [3.76]
RTs (ms)	Different		1022 [243]	844 [277]	714 [129]	1031 [254]	847 [271]	709 [138]
	Same	Identical	826 [269]	677 [213]	591 [77]	828 [227]	680 [207]	607 [86]
	Same	Mirror	826 [216]	705 [230]	625 [86]	807 [195]	707 [233]	620 [79]
	Same	Rotation	837 [179]	741 [236]	641 [80]	850 [191]	752 [260]	632 [71]

*Standard deviations in brackets*.

The final samples included 17 illiterates (12 women), aged 31–74 years (*M* = 56.6), 15 late literates (11 women), aged 19–71 years (*M* = 49.3), and 15 early literates (10 women), aged 27–68 years (*M* = 52.5). Early literates had on average 8 years of schooling (*SD* = 3.1). Illiterates were either recruited through non-governmental agencies or were attending the first lessons (first 2 weeks) of literacy classes, during which they received only information about civil rights and possible courses. Late literates were engaged in or already had finished the fourth (final) level of the literacy course. The three groups were from the same socioeconomic and residential backgrounds and had similar ages, *F* < 1.

All participants were first presented with letter recognition and reading (6 words and 6 pseudowords) tests. Illiterates were able to identify, on average, 8.65 letters out of the 23 letters of the Portuguese alphabet, and only one of them was able to read a single word (*M* = 0.49%). Almost all late literates correctly identified the 23 letters (*M* = 22.67) and reached at least 83.3% correct in the reading test (*M* = 95.6%). Except for one participant who did not recognize one letter, all early literates were perfect in both the letter recognition (*M* = 22.93) and the reading (*M* = 100%) tests. In the analyses of variance (*ANOVA*) on these scores, the main effect of group was significant on both letter recognition and reading performance, *F*_(2, 44)_ = 88.88 and = 3052.46, respectively, both *p* < 0.0001^1^. *Post-hoc* tests^2^ showed that late and early literate adults presented the same level of performance on letter recognition, both differing from illiterates, both *p* < 0.01. In the reading test, all groups differed from each other, *p* < 0.05 in all cases.

In order to evaluate potential cognitive differences, all participants were tested with the Mini-Mental State Examination (*MMSE*, Folstein et al., 1975). Because this test is known to be sensitive to educational and (correlated) literacy level (e.g., Crum et al., 1993), we used MMSE revised scores, recalculating individual scores after discarding the three items that examine reading, writing, and arithmetic abilities. This led to similar mean scores of 23.47 (*SD* = 3.02), 22.47 (*SD* = 1.77), and 23.33 (*SD* = 1.68) by illiterates, late literates and early literates, respectively, *F* < 1.^3^

After the orientation-independent tasks presented here, 38 participants (12 illiterates, 13 late literates, and 13 early literates) were also tested on orientation-dependent tasks using either pictures of familiar objects or geometric shapes (for detailed method and results, see Fernandes and Kolinsky, 2013). In the orientation-dependent task, the illiterates who were presented with both types of tasks showed difficulties especially in discriminating mirror images, obtaining 64.8% correct on “different” trials involving mirror images (64.17% for familiar objects, 65.5% for geometric shapes) vs. more than 80% correct on “different” trials involving plane rotations (82.1% for familiar objects, 80.3% for geometric shapes) and more than 85% correct on “same” trials (85.8% for familiar objects, 86.8% for geometric shapes).

#### Material and procedure

**Figure 1:**
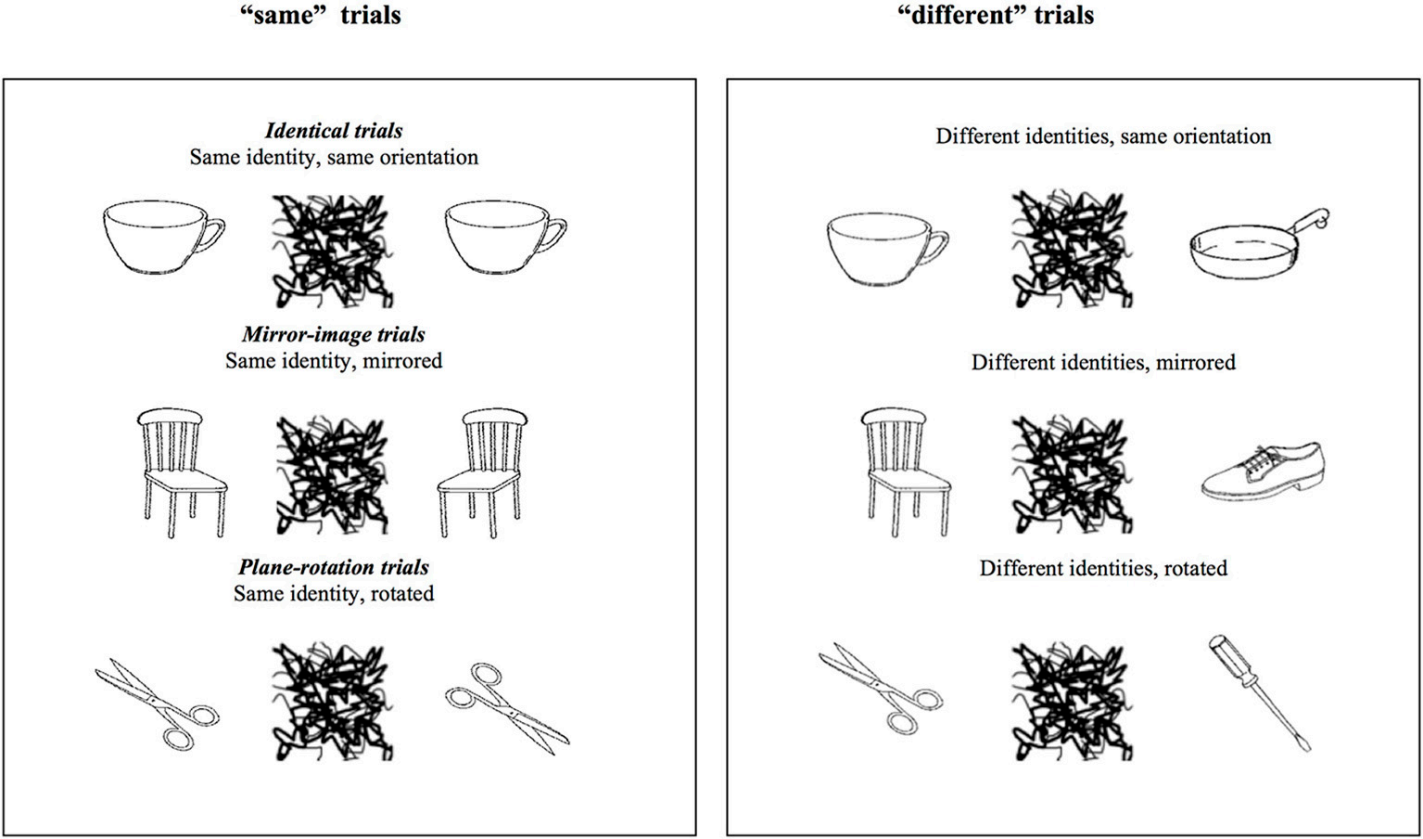
**Examples of the stimuli used in the “same” and “different” trials of Experiment 1**. The critical trials are the three types of “same” trials.

Stimuli were black and white pictures of asymmetric real objects. As explained in detail in Fernandes and Kolinsky (2013), most were from Snodgrass and Vanderwart (1980), the others were from Bonin et al. (2003). Examples are presented in Figure 1.

A total of 36 different objects (see the Appendix in Fernandes and Kolinsky, 2013) was used, half being graspable, the others non-graspable, as assessed by an independent group of participants (see Fernandes and Kolinsky, 2013). According to the norms collected by Ventura (1980), the two categories were matched on visual ambiguity, complexity, and familiarity, all *t* < 1.

For each object, a standard position, corresponding always to S1, was defined, and for the S2 a mirror image (lateral reflection) as well as a plane rotation were created, both differing from the standard by 180°.

Each trial started with a fixation cross presented in the center of the screen for 250 ms, after which S1 was presented during 2000 ms, then a 500 ms mask comprising random lines separated the presentation of S2 from the presentation S1 in order to guarantee no involvement of iconic memory in performance. On each trial, participants were asked to decide as quickly and as accurately as possible whether the second object was the same or not as the first, independently of its orientation. They thus had to answer “same” if S2 had the same identity as S1, independently of whether it had the same orientation (identical trials) or not, and to answer “different” if S2 had a different identity compared to S1, also independently of their orientation. As illustrated in Figure 1, on different-orientation trials, S2 could be either a mirrored or plane-rotated version of S1. RTs were measured from the onset of S2 to response onset. Immediately after participants gave their response another trial began, or if no response was provided the next trial began after 4750 ms.

Participants were presented with 864 trials, half “same,” half “different.” Each of the six possible pairs used for a particular object (see Figure 1) was presented twice, in different blocks. Participants were first presented with six practice trials to familiarize them with the task. They received feedback on the correctness of their response only for these trials.

### Results

Accuracy and RTs for correct responses were analyzed separately. For each participant, correct RTs longer or shorter than the grand mean plus or less 2.5 SD were removed from further analyses (less than 3% of the data excluded). In all analyses, RTs for correct responses were logarithmically transformed and accuracy was arcsine transformed^4^. Still, for the sake of clarity tables and figures present RTs in ms and accuracy in percentages.

Table 1 presents the mean scores for all trial types, separately for each group. Only the trials in which object identity was the same were considered in the following analyses. For both RTs and accuracy, we compared performance on physically identical trials to performance on trials in which object identity was also the same but where S2 was either a mirror image or a plane rotation of S1.

In a first step, we performed two separate ANOVAs, one on RTs, the other on accuracy, each with group (illiterates; late literates; early literates) as a between-participants variable and orientation (identical; mirror; rotation) and graspability (graspable vs. non-graspable objects) as within-participants variables.

There was a main effect of group for both RTs, *F*_(2, 44)_ = 6.79, *p* = 0.003, η^2^_*p*_ = 0.236, and accuracy, *F*_(2, 44)_ = 11.16, *p* < 0.001, η^2^_*p*_ = 0.337. *Post-hoc* comparisons showed that illiterates were significantly less accurate and slower than early literates, both *p* < 0.005, and less accurate, *p* = 0.003, but not slower, *p* = 0.10, than late literates, whereas late and early literates did not differ from each other in either analysis, both *p* > 0.30.

No other significant effect was found in the accuracy analysis, all other *F* < 1, including the main effects of orientation and of graspability, and the orientation by group interaction. Graspability did not affect performance on RTs either, *F* < 1.

Yet, orientation strongly affected performance in the RTs analysis, *F*_(2, 88)_ = 27.31, *p* < 0.001, η^2^_*p*_ = 0.383, in which its effect was modulated by group, *F*_(4, 88)_ = 2.48, *p* < 0.05, η^2^_*p*_ = 0.101. Orientation of the stimulus strongly affected the response speed of both late literate, *F*_(2, 28)_ = 35.27, *p* < 0.001, η^2^_*p*_ = 0.716, and early literate adults, *F*_(2, 28)_ = 13.48, *p* < 0.001, η^2^_*p*_ = 490. In contrast, it only slightly and non-significantly modulated the illiterates' response latencies, *F*_(2, 32)_ = 2.35, *p* = 0.11, η^2^_*p*_ = 0.111. Whereas illiterates' responses to mirrored trials were as fast as those to identical trials, *F* < 1, in the two literate groups, performance was slower for mirror images compared to identical trials [late literates: *F*_(1, 28)_ = 9.83, *p* = 0.004; early literates: *F*_(1, 28)_ = 10.56, *p* = 0.003]. For rotations, all groups presented slower responses compared to both identical trials [illiterates: *F*_(1, 44)_ = 3.95, *p* = 0. 05; late literates: *F*_(1, 28)_ = 49.95, *p* < 0.001; early literates: *F*_(1, 28)_ = 22.89, *p* < 0.001] and mirror images [illiterates: *F*_(1, 44)_ = 15.32, *p* < 0.005; late literates: *F*_(1, 28)_ = 32.26, *p* < 0.001; early literates: *F*_(1, 28)_ = 6.15, *p* = 0.019].

**Figure 2:**
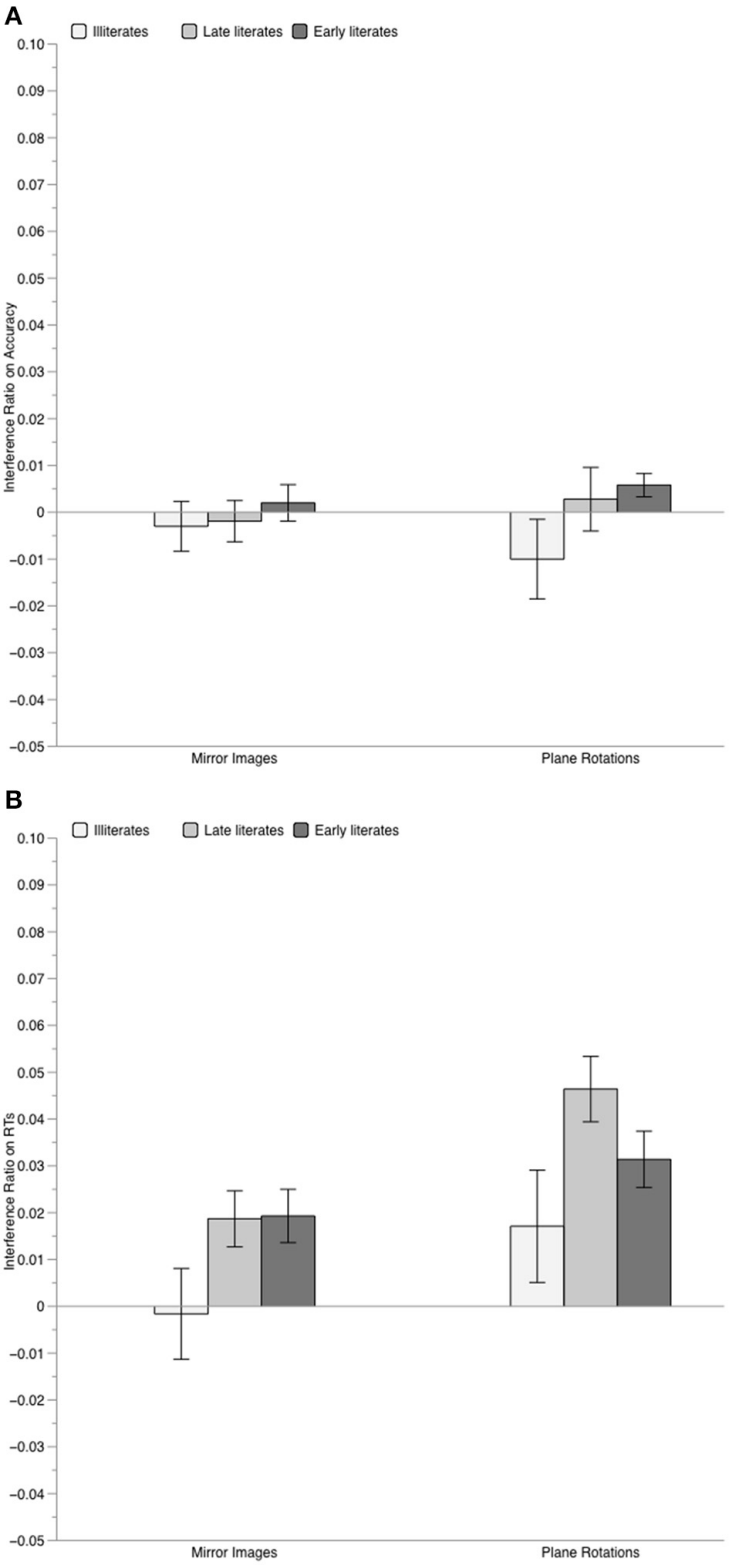
**Mean value of the interference index for familiar objects, computed on accuracy scores (Panel A) and on RTs (Panel B), separately for each group of participants**. Error bars represent standard error of the mean.

The analyses of the interference indexes (performed without taking graspability into account, as this factor did not affect performance) showed, in addition, that illiterates were less susceptible to irrelevant orientation variations than literates for both mirror images and (although to a lesser extent) for rotations. As illustrated in Figure 2, on the RT interference index, only illiterates were unaffected by orientation variations, with both mirror interference and rotation interference not differing from zero, *t* < 1 and *t*_(16)_ = 1.39, *p* = 0.18, respectively. In contrast, both literate groups presented significant mirror interference [late literates: *t*_(14)_ = 3.00, *p* = 0.009; early literates, *t*_(14)_ = 3.41, *p* = 0.004] and rotation interference [late literates: *t*_(14)_ = 6.63, *p* < 0.001; early literates: *t*_(14)_ = 5.16, *p* < 0.001]. On the accuracy interference index, only early literates showed significant rotation interference, *t*_(14)_ = 2.33, *p* = 0.035, all other *p* > 0.20.

Since the size of interference was similar for late and early literates for both mirror images, *t* < 1, and plane rotations, *t*_(28)_ = 1.61, *p* = 0.12, we contrasted the illiterate group to these literate participants. Compared to them, illiterate adults clearly presented weaker mirror interference, *t*_(45)_ = −2.27, *p* = 0.028, and somewhat weaker rotation interference, *t*_(45)_ = −1.96, *p* = 0.056.

### Discussion

Our previous work had shown that breaking mirror generalization depends on literacy acquisition in the Latin alphabet (Kolinsky and Verhaeghe, 2011; Kolinsky et al., 2011; Fernandes and Kolinsky, 2013). Here, similarly to former studies (Dehaene et al., 2010a; Pegado et al., 2011), we demonstrated that in adult readers enantiomorphy is automatically evoked during object recognition. In addition, confirming the results reported by Pegado et al. (2014), we showed that this process is a consequence of literacy acquisition: in an identity-based same-different comparison task in which participants had to respond “same” to both physically identical and differently oriented pictures of the same object, only literate but not illiterate adults were affected by irrelevant enantiomorphic variations. Thus, in literates, breaking mirror invariance interferes with a non-linguistic object recognition task when orientation is neither relevant nor useful for it. Furthermore, as predicted by the neuronal recycling hypothesis (Dehaene and Cohen, 2007; Dehaene, 2009), rotation interference was stronger than mirror interference, at least in literates. Mirror-image contrasts thus remain less salient or less automatically evoked than plane rotations, when processing the identity of familiar objects, probably because enantiomorphy is learned in the course of literacy acquisition. However, contrary to our prediction, no effect of graspability was observed.

## Experiment 2—identity judgments on geometric shapes

### Method

#### Participants

Among the participants of Experiment 1, 46 participated in this experiment: 16 illiterates, and all the late and early literates. As in Experiment 1, we first checked for task commitment, examining the SDT *d*′ scores in the same-different comparison task. One illiterate who presented a *d*′ ~ 0 was excluded from further analyses. All other participants were able to correctly perform the task with mean *d*′ scores of 3.95 (*SD* = 1.93), 4.92 (*SD* = 0.99), and 5.17 (*SD* = 0.92) by illiterates, late and early literates, respectively.

The final illiterate sample thus included 15 participants (10 women), aged 31–74 years (*M* = 56.0). They were able to identify, on average, 8.3 letters out of the 23 letters of the Portuguese alphabet, and none was able to read a single word of the reading test. Their mean revised MMSE score was 23.80 (SD: 3.14; same score as the unrevised one).

#### Material and procedure

**Figure 3:**
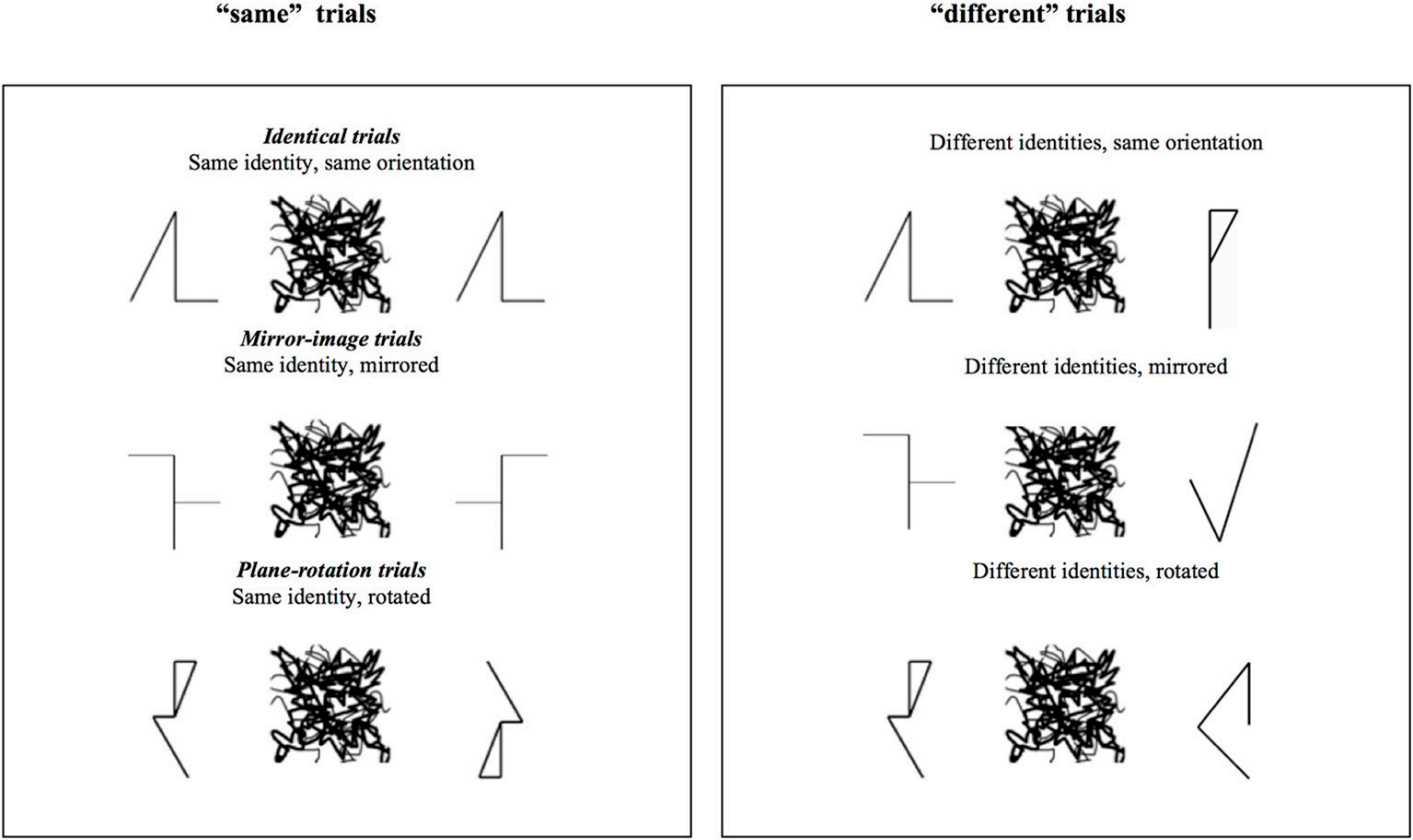
**Examples of the stimuli used in the “same” and “different” trials of Experiment 2**. The critical trials are the three types of “same” trials.

Nine asymmetric geometric shapes were used as S1 (see examples in Figure 3).

Construction of the pairs and trial types were identical to Experiment 1 (see Figure 3). Participants were presented with a total of 216 trials, half “same,” half “different.” Each S1 shape was paired four times with a replica and four times with its mirror image and with its plane rotation. For “different” trials, each S1 shape was paired four times with a different geometric shape, with a mirror image, and with a plane rotation of that shape.

Procedure was the same as in Experiment 1.

### Results

Data were trimmed (<3% of data excluded) and analyzed as in Experiment 1. Table 2 present the mean scores for all trial types, separately for each group.

**Table 2:** **Experiment 2: Mean performance in the identity-based same-different comparison task for geometric shapes, presented by trial type and group of participants**.

%	**Trial type**				
	**Expected response**	**Orientation**			
Accuracy (%)	Different		80.17 [20.08]	92.09 [7.56]	94.13 [4.68]
	Same	Identical	83.67[19.63]	95.00 [5.24]	95.80 [7.16]
	Same	Mirror	83.67 [15.67]	91.53 [7.69]	92.27 [6.24]
	Same	Rotation	80.87 [19.14]	88.53 [10.12]	90.67 [9.62]
RTs (ms)	Different		1194 [301]	960 [232]	836 [218]
	Same	Identical	941 [322]	734 [138]	723 [136]
	Same	Mirror	1034 [375]	800 [155]	747 [155]
	Same	Rotation	1055 [304]	863 [211]	815 [168]

*Standard deviations in brackets*.

**Figure 4:**
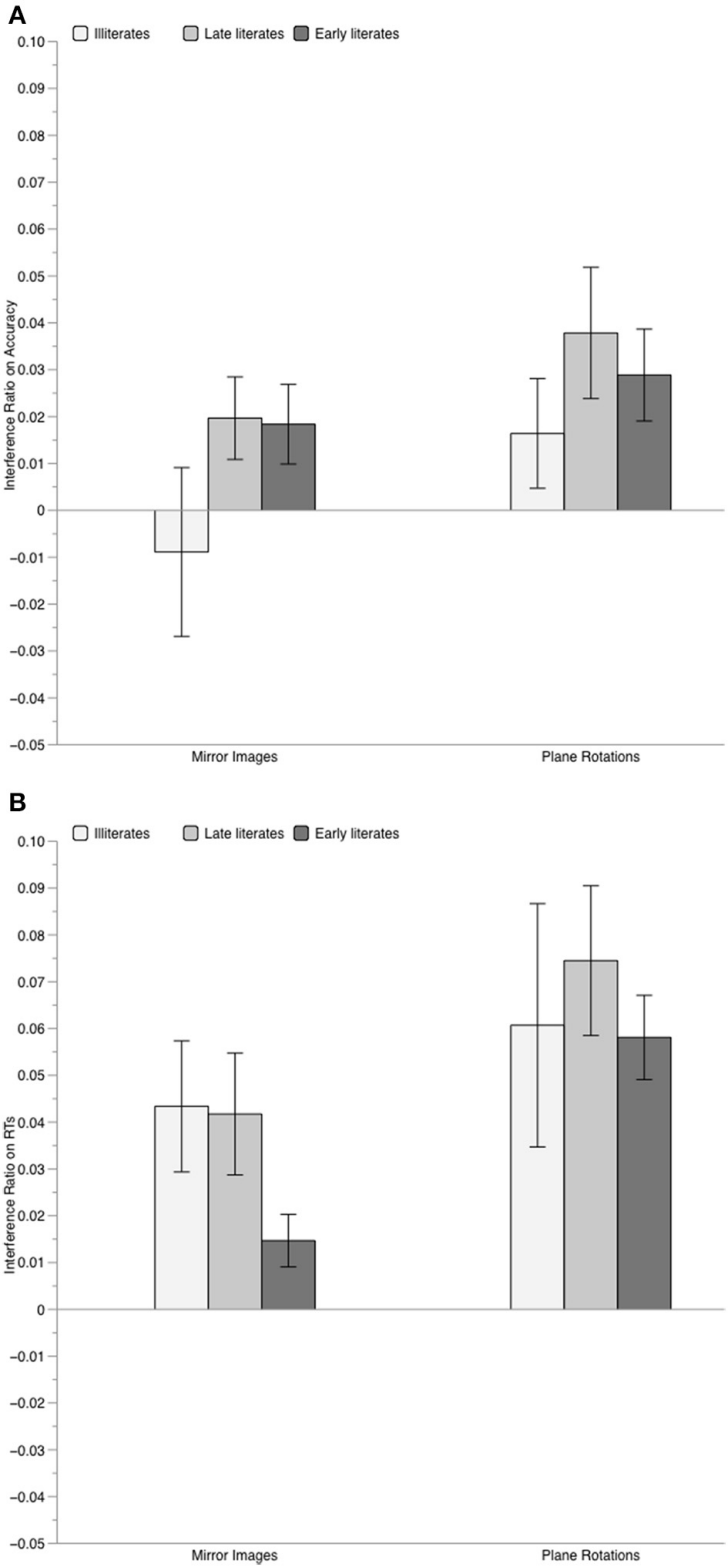
**Mean value of the interference index for geometric shapes, computed on accuracy scores (Panel A) and on RTs (Panel B), separately for each group of participants**. Errors bars represent standard error of the mean.

In the ANOVA on accuracy, only the main effect of orientation was significant, *F*_(2, 84)_ = 14.83, *p* < 0.001, η^2^_*p*_ = 0.261, with identical trials leading to better performance than both mirror images, *F*_(1, 42)_ = 12.43, and rotations, *F*_(1, 42)_ = 25.59, both *p* ≤ 0.001 (mirror images vs. rotations: *F* = 3.79, *p* = 0.058). The group effect only tended toward significance, *F*_(2, 42)_ = 2.87, *p* = 0.068, η^2^_*p*_ = 0.120. Although the interaction between group and orientation was not significant, *F* = 1.2, we further examined the effect of orientation on performance of each group, considering both the results of Experiment 1 and prior results on literate participants showing that they are more sensitive to orientation variations than illiterates (Pegado et al., 2014). In fact, whereas no effect of orientation was found in illiterates, *F*_(2, 28)_ = 1.76, *p* = 0.19, the effect of orientation was significant for both late literates, *F*_(2, 28)_ = 6.82, *p* = 0.003, and early literates, *F*_(2, 28)_ = 9.17, *p* < 0.001. In the two literate groups, relative to identical trials, performance was worse for mirror images [late literates: *F*_(1, 14)_ = 5.32, *p* = 0.036; early literates, *F*_(1, 14)_ = 12.36, *p* = 0.003], and for plane rotations [late literates: *F*_(1, 14)_ = 10.36, *p* = 0.006; early literates: *F*_(1, 14)_ = 12.11, *p* = 0.001]. Consistently, the analyses of the accuracy interference indexes (see Figure 4A) showed that only the literates were penalized by orientation variations, with significant mirror interference [late literates: *t*_(14)_ = 2.22, *p* = 0.043; early literates: *t*_(14)_ = 2.14, *p* = 0.049] and rotation interference [late literates: *t*_(14)_ = 2.77, *p* = 0.015; early literates: *t*_(14)_ = 2.94, *p* = 0.010]. In contrast, illiterates exhibited no mirror interference, *t* < 1, nor rotation interference, *t*_(14)_ = 1.40, *p* = 0.18. Since the amount of mirror and rotation interference was similar for late and early literates, both *t* < 1, we tested whether illiterates presented weaker interference than the literate participants. This was the case for mirror interference, *t*_(42)_ = −1.80, *p* = 0.038, but not for rotation interference, *t*_(42)_ = −1.18, *p* = 0.122.

Yet, the RT analysis suggested that even illiterates were somewhat sensitive to irrelevant mirror images of geometric shapes: both the main effect of group, *F*_(2, 42)_ = 5.02, *p* = 0.01, η^2^_*p*_ = 0.193 (with illiterates overall slower than late and early literates, *p* < 0.05, and *p* = 0.01, respectively), and of orientation, *F*_(2, 84)_ = 26.8, *p* < 0.001, η^2^_*p*_ = 0.389, were significant, but not their interaction, *F* < 1. Contrary to what was observed on accuracy, the effect of orientation was significant in all groups [illiterates: *F*_(2, 28)_ = 4.56, *p* = 0.02; late literates: *F*_(2, 28)_ = 14.45, *p* < 0.001; early literates: *F*_(2, 28)_ = 24.83, *p* < 0.001]. Across groups, performance was the slowest for rotations compared to identical trials, *F*_(1, 42)_ = 36.54, and to mirror images, *F*_(1, 42)_ = 13.14, both *p*s < 0.001, and was also slower for mirror images than for identical trials, *F*_(1, 42)_ = 24.80, *p* < 0.001. Thus, in terms of latency both illiterate and literate participants displayed mirror and rotation interference. The same conclusion can be drawn from the analysis of the RT interference index: as illustrated in Figure 4B, mirror and rotation interference effects were significant in all three groups (all *p* ≤ 0.03). No difference between illiterate and literate participants was observed, neither for mirror interference, *t*_(43)_ = 1.05, *p* = 0.300, nor for rotation interference, *t*_(43)_ = −0.25, *p* = 0.803.

### Discussion and cross-experiments analyses

Contrary to what was observed in Experiment 1 with familiar objects, here with geometric shapes all participants, whatever their literacy level, were sensitive to the irrelevant orientation variations, at least on response latencies and mostly for plane rotations.

To check for the robustness of this material difference, we performed cross-experiment analyses on the accuracy and RT interference indexes of the 43 participants (13 illiterates, 15 late literates, 15 early literates) who were presented with both materials and adequately performed the identity-based task. There was a significant main effect of material in both analyses, accuracy, *F*_(1, 40)_ = 10.31, *p* = 0.003, η^2^_*p*_ = 0.205, RT, *F*_(1, 40)_ = 8.37, *p* = 0.006, η^2^_*p*_ = 0.173, with an overall stronger interference effect with geometric shapes than with familiar objects. The main effect of orientation was also significant in both analyses, accuracy, *F*_(1, 40)_ = 7.04, *p* = 0.01, η^2^_*p*_ = 0.150, and RT, *F*_(1, 40)_ = 24.42, *p* < 0.001, η^2^_*p*_ = 0.379, with overall stronger rotation than mirror interference. The interaction between material and orientation was only significant in accuracy, *F*_(1, 40)_ = 7.68, *p* = 0.008, η^2^_*p*_ = 0.161, not on RTs, *F* < 1: rotation interference was stronger with geometric shapes than with familiar objects, *F*_(1, 40)_ = 17.64, *p* < 0.001, whereas mirror interference was similar with both materials, *F*_(1, 40)_ = 1.77, *p* = 0.191. In neither analysis did group interact with any other factor, all *p*s > 0.10. Thus, in comparison to familiar objects, identity-based judgments on geometric shapes were more strongly affected by irrelevant plane rotations, whatever the literacy level of the participant.

Given that 38 of the participants of the present study had also performed orientation-dependent tasks with the same materials (Fernandes and Kolinsky, 2013), we next examined whether there was any association between the interference effects reported here and the performance level observed for either mirrored or rotated trials in the orientation-dependent tasks by Fernandes and Kolinsky (2013). Across materials, no correlation was observed between this performance and the RT interference index, all *rs < 0.195*, *p*s > 0.24, but when accuracy was considered, there was a significant correlation between enantiomorphic performance and mirror interference, *r*_(36)_ = 0.387, *p* = 0.016, but not between plane rotation discrimination and rotation interference, *r*_(36)_ = −0.176, *p* = 0.289. Thus, the better the participants discriminated mirror images, the stronger these interfered on their identity-based judgments.

## General discussion

Literacy is an acculturation process that enables massive cognitive gains. However, according to the neuronal recycling hypothesis (Dehaene and Cohen, 2007; Dehaene, 2009), this new cultural ability may compete with evolutionary older functions, leading to collateral effects. As a matter of fact, enantiomorphy, namely the ability to discriminate between mirror images that develops through reading acquisition (Kolinsky and Verhaeghe, 2011; Kolinsky et al., 2011; Fernandes and Kolinsky, 2013), collides with the original mirror invariance property of the ventral visual system. Therefore, in the present study we investigated whether enantiomorphy interferes with object identity judgments, as suggested by former work (Dehaene et al., 2010a; Pegado et al., 2011, 2014). In particular, we examined whether the expected mirror interference reflects a specific impact of literacy on enantiomorphy rather than a general impact on orientation processing during object recognition. Furthermore, we also checked whether the strength of the interference displayed by illiterate and literate adults would be modulated by the familiarity of the material and, for familiar objects, by their graspability (Fernandes and Kolinsky, 2013). To these aims we presented illiterate, late literate (who learned to read at adult age) and early literate adults with an identity-based same-different comparison task in which they had to respond “same” to physically identical, mirrored, and plane-rotated images of either pictures of familiar objects (Experiment 1) or geometric shapes (Experiment 2). We examined the interference from irrelevant orientation variations separately for mirror images and plane rotations.

With pictures of familiar objects, contrary to literate adults, illiterates did not display any mirror interference. As expected, for all groups, interference was stronger with geometric shapes than with familiar objects. With geometric shapes, both plane rotations and enantiomorphic variations affected response latencies, irrespective of the participants' literacy level. Still, in terms of accuracy, contrary to literates, illiterates did not display mirror interference with geometric shapes, whereas they did show rotation interference.

In what regards familiar objects' graspability, namely the degree by which visuomotor information is critical to the representation of the object, in contrast to our prediction, this property had no impact on identity-based judgments. This result pattern stands in sharp contrast to that found by Fernandes and Kolinsky (2013) in an orientation-dependent task. There, the explicit discrimination of orientation variations, either mirror images or plane rotations, was facilitated for graspable objects. Note, however, that the orientation variations that could have invoked action-related information of graspable objects were in the present study irrelevant to the task. Prior studies have shown that the visuomotor properties of objects are especially processed by the dorsal, vision-for-action stream (e.g., Valyear et al., 2006; Rice et al., 2007). In particular, parietal regions, part of the dorsal stream, have been shown to be critical for processing spatial attributes of objects in orientation-based tasks, but not their identity (Harris et al., 2008). Therefore, although both ventral and dorsal streams operate simultaneously during visual processing, their relative involvement depends on the specific task. Task specificities might thus explain the apparent discrepancy between the graspability effects found in the orientation-based task used by Fernandes and Kolinsky and their absence in the identity-based task of the present study. Further brain-imaging studies could test this possibility.

More importantly, the present result pattern is in line with prior studies showing that the discrimination of mirror images and of plane rotations are supported by at least partially different mechanisms (e.g., Turnbull et al., 1997; Turnbull and McCarthy, 1997), and that the ventral visual pathway is originally sensitive to plane rotations but not to mirror images (e.g., Logothetis and Pauls, 1995). In this vein and in line with our prediction, across groups and experiments, plane rotations interfered more on identity judgments than mirror images. Furthermore, it was only for mirror images that the size of the interference effect was linked to the participants' enantiomorphic performance in an orientation-dependent task (cf. Fernandes and Kolinsky, 2013): the better they could discriminate mirror images, the stronger the mirror interference on their identity-based judgments.

The process of unlearning mirror invariance, necessary to acquire literacy in the Latin alphabet, has thus a cost for object identification, a basic function of the visual ventral stream. The observation of a negative side effect of a literacy-related ability, namely enantiomorphy, was expected under the neuronal recycling hypothesis (Dehaene and Cohen, 2007; Dehaene, 2009), which proposes that reading, as other recent cultural inventions, capitalizes on evolutionary older functions, with which they may compete. Brain-imaging data had already shown that literacy induces a profound reorganization of the cortical networks for vision and language, and that this process involves competition for neural space in the left fusiform gyrus, especially between written strings and faces (Dehaene et al., 2010b).

A functional cost like the one reported here is also expected if some properties that were useful for the original function are deleterious for the new function, and hence, should be unlearned. As a direct consequence, this unlearning process would benefit the new function (here, reading) but harm the older one. Effects of both neural competition (Dehaene et al., 2010b) and functional competition as shown here, as well by Dehaene et al. (2010a) and Pegado et al. (2011, 2014), thus demonstrate that neural recycling is not just an adaptation to multi-use (see discussion in, e.g., Jungé and Dennett, 2010) but a process of at least partial exaptation. More generally, as noted by Dehaene (2013), the presence of mirror invariance prior to literacy and its reduction during reading acquisition show that learning to read involves the recycling of a preexisting circuit that did not evolved purposely for reading, but adapts to this novel task.

### Conflict of interest statement

The authors declare that the research was conducted in the absence of any commercial or financial relationships that could be construed as a potential conflict of interest.

## Footnotes

^1^As usual, for all inferential statistics presented in this study, *p* < 0.05 is interpreted as a statistically significant result.

^2^All *post-hoc* between-group tests reported in the present study correspond to unequal N HSD tests.

^3^As expected, when the items examining reading and writing abilities were also taken into account, the group effect became significant, *F*(2, 44) = 20.97, *p* < 0.0001, with illiterates differing from both late and early literates (*M* = 23.47, 27.47, and 28.33, respectively), both *p* < 0.001.

^4^Given that proportions usually follow a binomial distribution in which the variance is a direct function of the mean, the arcsine transformation allowed guaranteeing no violation of the normality assumption necessary for conducting parametric analyses (e.g., Howell, 2010).
